# Unified climate factors predict influenza outbreak seasonality across tropical and temperate regions

**DOI:** 10.1093/pnasnexus/pgag160

**Published:** 2026-06-16

**Authors:** Aleksandra R Stamper, Wenchang Yang, Caroline E Wagner, Ayesha S Mahmud, Rachel E Baker

**Affiliations:** Department of Epidemiology, Brown University School of Public Health, 121 S Main Street, Providence, RI 02903, USA; Institute at Brown for Environment and Society, Brown University, 85 Waterman Street, Providence, RI 02912, USA; Department of Geosciences, Princeton University, Briger Hall, Princeton, NJ 08544, USA; Department of Bioengineering, McGill University, 3480 Rue University 350, Montreal, Quebec H3A 0E9, Canada; Department of Demography, University of California, Berkeley, Berkeley, CA 94720, USA; Department of Epidemiology, Brown University School of Public Health, 121 S Main Street, Providence, RI 02903, USA; Institute at Brown for Environment and Society, Brown University, 85 Waterman Street, Providence, RI 02912, USA

**Keywords:** Influenza, climate change, SIR model, simulation

## Abstract

Influenza represents a source of considerable morbidity and mortality worldwide, exhibiting seasonal outbreak patterns that vary by location. Temperate regions typically experience sharp wintertime peaks, while tropical regions tend to exhibit lower-intensity, year-round activity, sometimes with two outbreak peaks a year. A key question is how a common set of factors interact to produce distinct outbreak patterns across temperate and tropical locations. Here, we compile a novel dataset of influenza surveillance data across North and South America and fit a mechanistic susceptible–infected–recovered–susceptible model driven by locally resolved climate data to test whether a unified set of climate variables can explain regional differences in influenza outbreak dynamics. We find that a combined, nonlinear effect of specific humidity and temperature can explain the seasonality of outbreaks across both temperate and tropical climate regimes. Leveraging this model, we explore the potential impacts of climate change on influenza outbreaks. We find that while temperate areas may experience a decline in peak size, outbreak intensity in tropical areas could increase under climate change.

Significance statement Influenza epidemics occur worldwide but vary in timing and intensity across climates. Temperate regions experience intense wintertime influenza outbreaks, while tropical areas exhibit a wider range of activity including year-round infections. We fit a climate-driven epidemiological model to 81 sites across the Americas, finding a common nonlinear effect of climate on transmission is able to predict influenza activity across temperate and tropical regions. Estimating transmission as a function of climate allows us to simulate future influenza outbreaks under climate change projections. We find that outbreak intensity may decline in temperate regions but increase in tropical areas over the next century. This study provides a unified explanation for the role of climate in shaping outbreaks across latitudes.

## Introduction

Seasonal influenza imposes a significant global public health burden each year, with an estimated 1 billion cases, including 3–5 million cases of severe illness and up to 650,000 annual deaths worldwide (1). In temperate regions, influenza exhibits annual wintertime epidemics, with out-of-phase outbreaks in the Northern and Southern hemisphere aligned with their respective winter months (2, 3). In contrast, influenza outbreaks in tropical regions appear to experience distinct dynamics, often with transmission persisting year-round (4–6) and peak activity occurring either twice annually or during the rainy season (6, 7). A large body of laboratory and observational evidence has identified specific humidity—the mass of water vapor per unit mass of air—as a key climate factor influencing influenza transmission, affecting viral particle survival (8), the timing of the seasonal epidemic onset (2), and broader outbreak characteristics (5). Although establishing a causal relationship between specific humidity and influenza activity is challenging given the complex, seasonally forced dynamics of infectious disease transmission (9–11), converging laboratory and observational evidence supports a central role for climate factors in shaping influenza activity (12–14).

In temperate climates worldwide, where wintertime specific humidity is consistently low, this relationship is well characterized: reduced specific humidity enhances viral viability and transmission, driving intense seasonal epidemics that deplete the size of the susceptible population (2, 5, 8, 14–17). By contrast, less experimental and mechanistic work has examined influenza transmission in tropical settings, where outbreaks are generally less intense, more temporally dispersed, and occur under warmer and more humid conditions. Correlational evidence suggests that a nonlinear, U-shaped relationship between specific humidity and influenza incidence could reconcile observed patterns across temperate and tropical settings (5, 7, 18); yet, this relationship has not been fully characterized within a transmission model. While the tropical mechanistic model developed by Yuan et al. proposes a U-shaped climate-transmission relationship, its ability to generalize to temperate settings has not yet been tested. As a result, it remains unclear whether a common functional relationship between climate and transmission can explain differences in the dynamics of influenza outbreaks in both temperate and tropical climate locations.

Here, we test whether a single climate-transmission relationship can explain influenza transmission across both temperate and tropical sites using a mechanistic framework. A challenge for influenza control is the evolution of the influenza virus’s surface proteins, a process known as antigenic drift, which contributes to ongoing loss of population immunity (19). Mechanistic epidemiological models can track changes to population immunity via pathogen evolution, infection, or the birth of new susceptible individuals into the population (20). In these models, seasonality in transmission is often attributed to climate factors. We begin by compiling a dataset of influenza surveillance data at the national and subnational scale for North and South American countries. The dataset spans tropical, subtropical, and temperate locations, including those with arid and continental climates. Given expected differences in reporting across locations, we first use a scale-invariant metric, epidemic intensity (EI), to characterize outbreak patterns across latitude. We then develop and fit an epidemiological influenza model to data from each location, where the fitting algorithm estimates the effect of specific humidity and temperature on transmission using locally resolved climate data (21). We combine estimates for the effect of climate on transmission across locations to test whether a common set of climate factors can explain dynamics across tropical and temperate regions. We then leverage our climate-driven epidemiological model to project the future effect of climate change on influenza outbreaks.

## Results

### Influenza dynamics across North and South America

**Figure 1:**
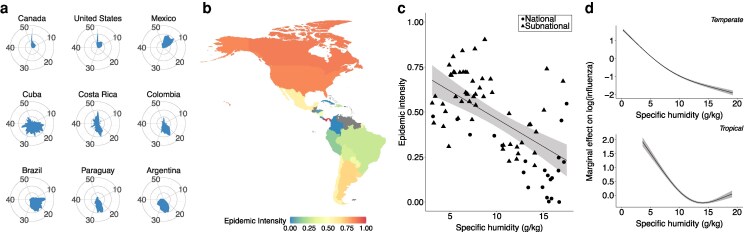
Influenza activity and characteristics across North and South American countries and states. a) Polar plots of scaled influenza time series (from 0 to 1) across select temperate and tropical countries; labels on the circular figure indicate week of the year. b) Country-level EI across North and South America. c) EI by mean specific humidity as averaged over all grid areas for each location, North and South American countries (circles) and states (triangles). Figure 2 analysis locations are labeled (Michigan, United States; Paraná, Brazil; Nicaragua). d) Generalized additive model regression results showing an approximately linear relationship between specific humidity and log influenza cases in temperate locations, and a nonlinear relationship in tropical locations (tropics defined as ±23.5${\;}^{\circ }$ latitude).

Figure 1a presents polar plots of average weekly influenza activity throughout the year, using national-level data from the World Health Organization’s Global Influenza Programme. In each polar plot, the circular axis represents the calendar year by week, allowing visualization of the seasonal distribution and timing of influenza activity. Temperate regions such as the United States, Canada, and Argentina display more concentrated wintertime outbreaks, while tropical countries such as Nicaragua or Colombia exhibit more prolonged and dispersed influenza activity throughout the year, consistent with year-round transmission. Certain tropical countries, such as Costa Rica, exhibit two peak periods of influenza activity, a phenomenon that has been noted in other tropical locations for influenza (7), and other directly transmitted viral pathogens (22).

We calculate influenza EI for all countries and sub-national locations in our compiled influenza surveillance dataset (Fig. 1b,c). EI is defined based on the Shannon entropy (see Methods (23)) and scaled between 0 and 1: higher values imply shorter, more concentrated outbreaks whereas low values imply prolonged, persistent outbreaks. Figure 1b displays the spatial distribution of EI at the country level, revealing that higher EI values were generally observed in temperate regions such as North America or southern South America, while lower values were more common in equatorial areas. Figure 1c plots EI at both national and sub-national administrative level 1 (state level) against average specific humidity. We find a significant negative association (P<<0.001) between EI and average specific humidity, with locations experiencing lower specific humidity—typically at higher latitudes—exhibiting higher EI values.

We also fit a generalized additive model (GAM) to examine the relationship between specific humidity and weekly influenza case counts Fig. 1d, controlling for location- and time-varying effects (see Supplementary text). We find distinct patterns of climate dependence in tropical and temperate locations: the tropical effect appears nonlinear, while the temperate effect decreases monotonically with increasing specific humidity. Importantly, these results represent the effect of climate on incidence, not transmission: we hypothesize that interactions between climate, transmission and susceptible dynamics could explain these differences even while a common effect of climate on transmission remains.

### Estimating climate drivers of transmission

**Figure 2:**
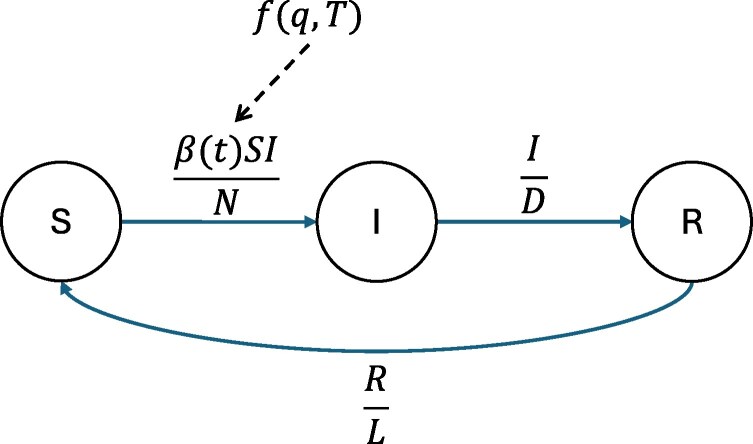
Compartmental SIRS model framework. Specific humidity, *q*, and temperature, *T*, drive the transmission rate *β*, *L* is the duration of immunity, and *D* is the duration of infection.

Figure 1 presents correlative evidence for a link between climate and influenza outbreaks, as described by EI and logged cases. In order to understand how climate mechanistically impacts transmission, we fit a climate-driven susceptible–infected–recovered–susceptible (SIRS) model to influenza data from each location in our dataset. The model was developed in prior work to study the drivers of influenza seasonality in tropical and sub-tropical locations and has not been tested in temperate locations (7, 18). The model allows transmission to be flexibly dependent on specific humidity and temperature, as represented in Fig. 2. We begin by fitting the model to three locations that represent a range of climate regimes: Michigan, United States; Paraná, Brazil; and Nicaragua. Michigan experiences a temperate climate, Nicaragua exhibits a tropical climate, while Paraná has a subtropical climate with a mix of temperate and tropical zones. We assume that nonclimatic factors such as vaccine efficacy, vaccine uptake, and strain circulation, may partially explain interannual differences in influenza outbreaks but that seasonal patterns are driven by climate: as such, we fit our model to weekly average scaled case counts (see Methods) (24). Latin hypercube sampling (LHS) is used to generate parameter sets sampled from ranges drawn from the literature (2, 18): we find the parameter set which minimizes the root mean-squared error between the simulated and observed cases (Fig. S1).

**Figure 3:**
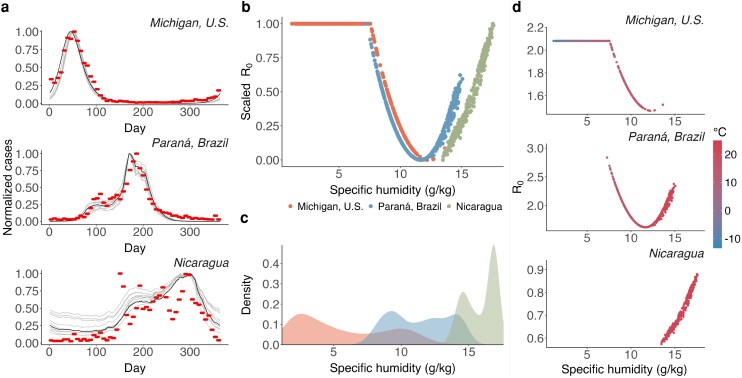
Fitted effect of climate on $R_{0}$ for Michigan, Paraná, and Nicaragua. a) Model fits for the three study locations: 10 best fit parameter combinations for each study location (lighter lines) with best fit (bold darker line) and scaled influenza data (points). b) Predicted average daily $R_{0}$ based on climate from best fit parameters for each location (vertically scaled). c) The kernel density plot shows specific humidity range for each location. d) Fitted dependence of average daily $R_{0}$ on specific humidity for each location, indicating temperature.

Figure 3a shows seasonal scaled incidence data (red points), best fit model (bold black line) and second through 10th best fits (gray lines). We find the model successfully recreates distinct seasonal patterns in all locations despite being previously only fit in tropical settings; for example, the model was able to recreate the wintertime outbreaks observed in Michigan, United States. Figure 3d shows the estimated effect of climate on transmission for each location. For Michigan, United States, the model estimates a negative relationship between specific humidity and transmission, which is supported by prior work in temperate locations (2); for Paraná, Brazil, the model estimates a U-shaped relationship between specific humidity and transmission, which is supported by prior work in tropical locations (4, 7); and for Nicaragua, the model estimates a positive relationship between specific humidity and transmission. We observe that the estimated effect of climate on transmission sketches out a complete curve across these three locations, albeit with different $R_{0}$ intercepts. In Fig. 3b, we combine the estimated effect, scaling $R_{0}$ values between 0 and 1 (while Fig. 3c shows the kernel density distribution of specific humidity values per site). The results from these three locations suggest that a common U-shaped relationship between specific humidity and $R_{0}$ can explain outbreaks across tropical and temperate regimes: this relationship is further modulated by temperature, with colder temperatures leading to increased transmission (Fig. 3d).

**Figure 4:**
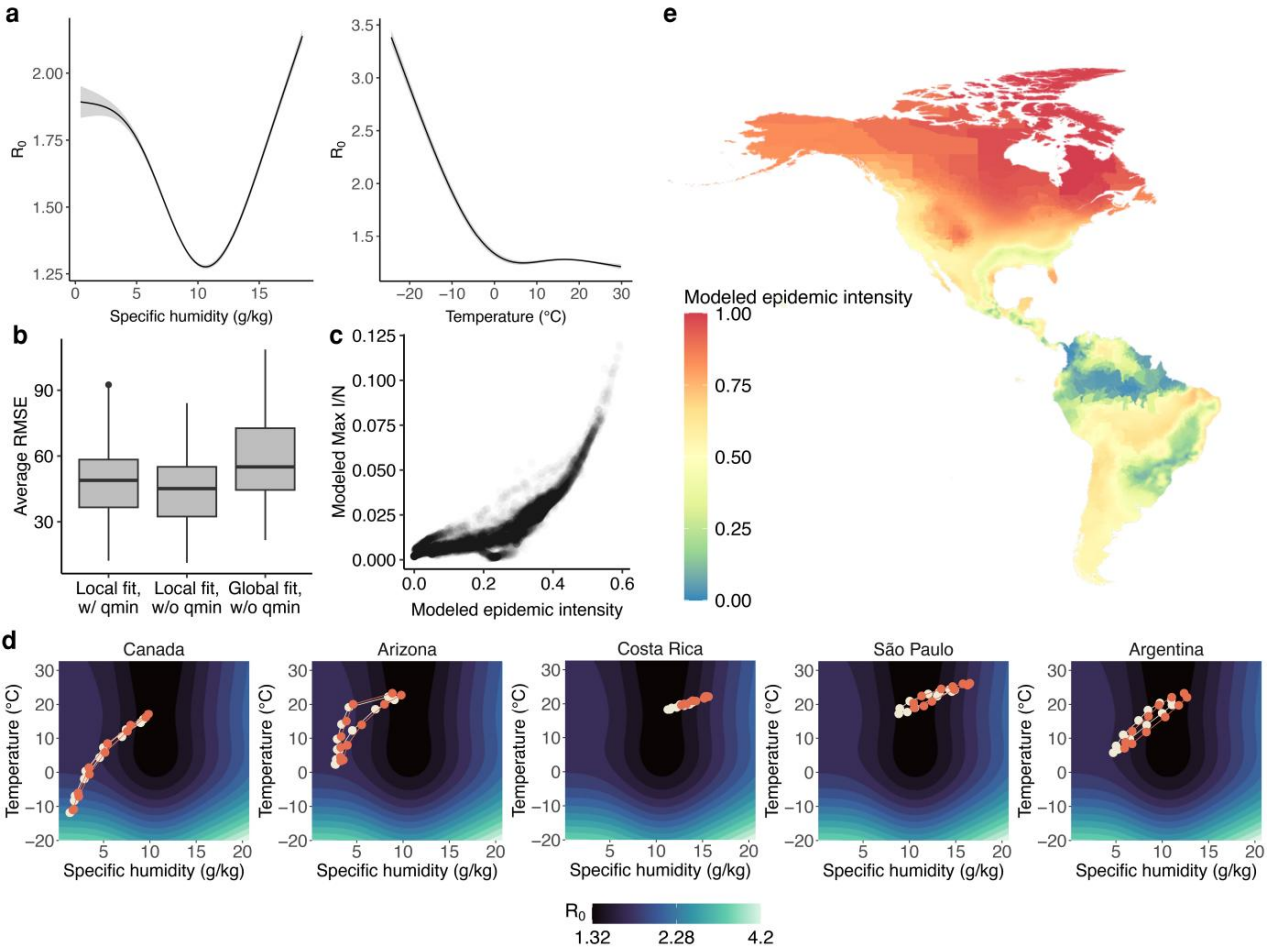
Pooled effect of climate on transmission across tropical and temperate locations. a) GAM indicating combined effects of specific humidity, q(t) (g/kg), and temperature, T(t) (C°), on influenza transmission. b) Assessing RMSE ranges during GAM optimization, where local fit indicates the best-fit for each individual location, and global fit indicates the best-fit scaling factor for all locations. c) Modeled relationship between EI and the maximum proportion infected, where larger infected peaks correspond to higher intensity. d) GAM-predicted effect of specific humidity and temperature on $R_{0}$. White connected dots indicate historic *monthly* ERA temperature and specific humidity average values, while the red connected dots display the same information under the average SSP 245 climate pathway per location. e) Model predicted EI values at the admin 2 (county) level using current climate averages (2000–2015).

Following the three initial locations (Michigan, Paraná, and Nicaragua), we next fit the model to 78 additional eligible locations based on inclusion criteria including data completeness (see Methods) and pool the resulting climate-transmission curves (see Fig. S2). To make the curves comparable across locations, we make two adjustments compared to the three-location analysis in Fig. 3. First, as opposed to scaling fitted $R_{0}$ values between 0 and 1, we retain the absolute range of fitted $R_{0}$ values and align values across locations based on overlapping means (see Supplementary text). Second, we remove the forced plateau at extreme specific humidity minima which was specified in the original model developed for the tropics (18) (see Michigan in Fig. 3c) but is not consistent with prior work on temperate regions (2, 8) (removing the plateau improves fit metrics, Fig. 4b).

As in our preliminary analysis, the aligned $R_{0}$ values suggest a U-shaped relationship between specific humidity and transmission, with both high and low specific humidity associated with elevated transmission risk (Fig. 3a). This U-shaped effect is asymmetric, with the highest transmission occurring under colder temperatures and low specific humidity. To capture these climate effects on $R_{0}$ in a single framework across all locations, we fit a GAM to the aligned location-specific $R_{0}$ estimates, using temperature and specific humidity as predictors (Fig. 3b). Figure 4a displays the predicted joint effects of specific humidity and temperature on transmission using the GAM model. In Fig. 4b, we compare alternative model specifications (see Supplementary text) including removing the specific humidity plateau and separately fitting a location-specific $R_{0}$ intercept term: we find the local $R_{0}$ intercept only improves fit marginally which provides support for using a global intercept when running models for out-of-sample locations and climate change projections.

We leverage the GAM-based estimates of climate effects on transmission as inputs to the mechanistic SIRS model, simulating outbreaks across locations in North and South America at the administrative unit 2 level. As shown in Fig. 4c, simulated EI (Fig. 4e) is strongly correlated with the maximum proportion infected. Figure 4d shows the predicted transmission surface based on specific humidity and temperature from our GAM model. We find that locations with wintertime outbreaks (eg Canada) experience increasing transmission with declining specific humidity, whereas locations summertime outbreaks in tropical areas (eg Costa Rica) experience increasing transmission as specific humidity increases. Our surface also predicts that certain locations may experience two periods of increase transmission a year, which explains the two annual influenza outbreaks in locations such as São Paulo (see Fig. S4).

### Projecting future outbreaks under climate change

**Figure 5:**
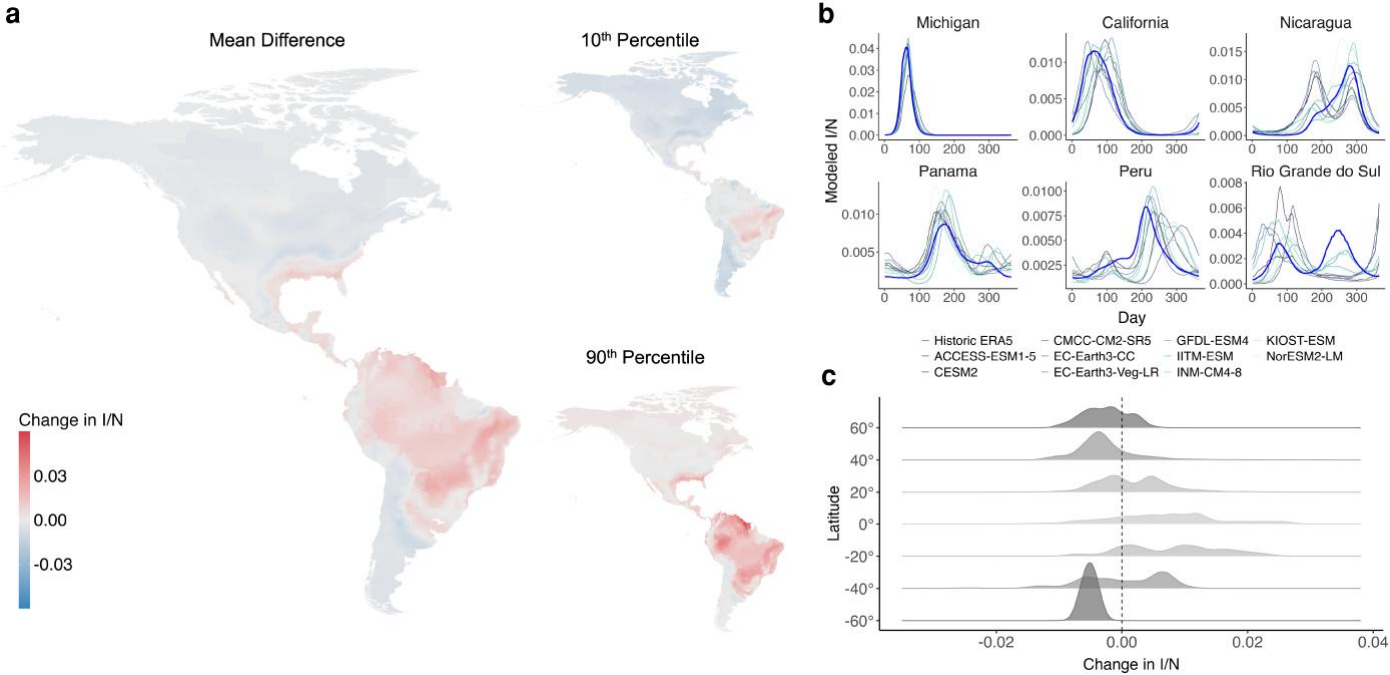
Simulated influenza outbreaks under historic and future climate projections. a) Estimated change in the epidemic peak size (max. proportion infected −  *I*/*N*) comparing historic (2000–2015) to mean, 10th percentile, and 90th percentile SSP245 projections for 2080–2100. b) Simulated outbreaks for select locations using historic (bold line) and projected (10 thin lines) climate data. c) Ridgeline plot showing change in peak size (Max. *I*/*N*) across locations categorized into 20${\;}^{\circ }$ latitude bins.

We leverage our climate-driven influenza model to understand the implications of climate change for influenza outbreaks. We take bias-corrected (see Methods) projected specific humidity and temperature data from 10 climate models contributing to Coupled Model Intercomparison Project Phase 6 (CMIP6) under Shared Socioeconomic Pathways 2 and 5 (SSP2 and SSP5) (25). The SSP2 projections represent a “middle-of-the-road” scenario with moderate climate mitigation, while the SSP5 reflects a high-emissions pathway with fossil fuel-intensive growth and limited climate policy (25). Figure 5a shows the projected mean change (future: 2080–2100 relative to historic: 2000–2015) in influenza epidemic peak size, as well as the 10th and 90th percentile change based on running our influenza model with input data from each climate model in the SSP2 pathway (Fig. S5 displays the same information for the SSP5 pathway). We find that for many temperate regions, climate change is projected to lead to a decline in the epidemic peak size of influenza outbreaks on average, whereas, for certain tropical regions, climate change may lead to an increase in epidemic peak size. This effect is driven by the predicted U-shaped relationship between specific humidity and transmission: as specific humidity increases, locations with on average lower specific humidity will experience a decline in transmission, whereas locations with on average higher specific humidity will experience an increase in transmission.

Figure 5b shows the simulated proportion infected for several locations in our dataset using input from each climate model. For locations with intense wintertime outbreaks, like Michigan, we do not observe substantial changes under climate change. We expect in these locations that the strong seasonal swing in temperature and humidity drives a steep wintertime peak in transmission and is not substantially changed under climate projections. However, for more tropical locations, we predict possible changes to both the size and timing of the outbreaks. For instance, in Nicaragua, certain climate models predict a more distinctly bimodal influenza outbreak under climate change. In contrast, in Rio Grande do Sul, bimodal outbreaks are on average replaced with a strong early influenza peak. The results suggest that climate change is most likely to affect influenza outbreaks in the tropics; in contrast, temperate locations may experience modest declines in epidemic peak size (Fig. 5c).

## Discussion

Our results demonstrate how a common set of climate factors, interacting through a shared functional form, can reproduce key features of influenza outbreaks across both tropical and temperate locations. In particular, a U-shaped dependence on specific humidity can explain wintertime outbreaks in temperate areas and summertime outbreaks in certain tropical locations, while a monotonically decreasing effect of temperature drives the more intense wintertime outbreaks in temperate regions. These findings align with prior statistical analyses of influenza seasonality that reported a U-shaped dependence on specific humidity with a minima at 11–12 g/kg (5); notably, the minimum of the specific humidity effect on transmission estimated by our GAM occurs at 11.58 g/kg. In addition, our results from the model-fit process estimate that temperatures above 23.1 ${\;}^{\circ }$C are detrimental to influenza particle survival, aligning with prior laboratory evidence suggesting that proteins in the bilayer nuclear envelope begin to denature around 21 ${\;}^{\circ }$C (26).

Building on these results, the functional form estimated in Fig. 2 extends prior climate-transmission frameworks by integrating temperature and specific humidity into a single, unified framework applicable across both temperate and tropical settings. Whereas previous studies applied similar formulations within limited geographic contexts (eg Yuan et al. and Mahmud et al.), we evaluate whether a unified climate-transmission framework can account for influenza dynamics across a broad set of temperate and tropical sites. Consistent with laboratory and observational evidence that low specific humidity can enhance influenza viral viability and transmission (8), the fitted surface exhibits decreasing transmissibility with increasing specific humidity up to the minimum of the U-shaped dependence. However, humidity alone is unlikely to account for influenza activity observed at higher levels in many tropical locations, motivating the inclusion of temperature as an additional predictor in the joint function f(q,T). At elevated specific humidity, lower temperatures may still promote transmission through their effects on viral viability, as the lipid envelope of the viral particle is more stable at lower temperatures (26). These complex effects are visualized in Fig. S3b, which presents a surface plot of predicted influenza transmission under the range of observed temperature and specific humidity combinations.

By directly linking climate variables to transmission, this framework also helps reconcile tropical-temperate differences in climate-incidence patterns (Fig. 1d). In temperate locations, large wintertime outbreaks fueled by low specific humidity and low temperature reduce the size of the susceptible population, meaning that summertime outbreaks are not observed, even if transmission increases due to high specific humidity. In contrast, outbreaks in tropical locations tend to be less intense, enabling sufficient replenishment of the susceptible population for two outbreaks a year to occur, during both high and low specific humidity periods.

EI is an entropy-based summary statistic, and its interpretation warrants consideration when comparing across populations and surveillance contexts. Under the Shannon formulation, intensity is the highest when cases are temporally concentrated and decreases as incidence becomes increasingly dispersed over a year (23). As a result, EI can be sensitive to noise in the underlying time series; however, this is less of a concern in our study, as the least populous state in the dataset—Acre, Brazil—has a population of ∼884,372 (27). Given the relatively large population even in the smallest state, we expect limited bias arising from locations with small population sizes. Additionally, our inclusion criteria were designed to ensure the influenza time series analyzed are minimally affected by sources of bias stemming from small population size or other reporting artifacts.

Pathogen evolution remains an important driver of influenza dynamics (28). Our model broadly captures evolution by allowing immunity to wane seasonally but does not capture specifics of strain dynamics. Prior work found that including co-circulation of multiple strains did not improve model performance for tropical locations (18). In our case, we assume that strain dynamics and vaccination (efficacy and uptake) may drive year-to-year differences in influenza outbreak size; as such, we fit our model to weekly mean case counts assuming that the mean seasonal outbreak pattern is dependent solely on climate factors. A limitation of our approach is that our model is only able to capture the present and projected component of influenza dynamics that is determined by climate. Future changes to vaccine efficacy, for example, the availability of a universal influenza vaccine (29), or changes to other factors associated with influenza outbreaks such as population density and mobility (23, 24, 30), will impact projections of outbreak patterns.

By relating climate variables to transmission, we develop a climate-driven mechanistic model for influenza that we leverage to predict influenza outbreak patterns across tropical and temperate regimes, as well as under future climates. Existing laboratory evidence suggests a negative relationship between specific humidity and influenza virus viability which explains outbreaks in temperate regions (2, 13). More recent work lays out a theoretical framework for a U-shaped dependence on relative humidity, with supporting experimental work on SARS-CoV-2 (31), but this effect has not been tested on population-level data. Future experimental work in climate-controlled environments with a focus on tropical-like climate conditions could help better elucidate the mechanistic link in these locations (32). More broadly, our work highlights that common environmental drivers can explain the outbreak dynamics of respiratory pathogens in a wide range of climate zones, recreating observed outbreak characteristics along a latitudinal gradient. Similar modeling approaches could be applied to other acute respiratory viral infections where latitudinal gradients are observed. Elucidating links with climate by leveraging large cross-national datasets could help improve scenario modeling and forecasting in locations where surveillance data is not available for certain pathogen types.

## Methods

### Influenza data

Data on weekly hospitalizations for influenza at the county level in the United States were obtained from the State Inpatient Databases (SID), sponsored by the Agency for Healthcare Research and Quality, part of the Healthcare Cost and Utilization Project (33). The SID includes data from all community hospitals at the county level between 1988 and 2010, covering 22 influenza seasons. Data on weekly influenza hospitalizations at the state level in Brazil from 2010–2022 were downloaded from the SIVEP-Gripe Data Repository, part of the Base dos Dados Project developed by the Ministry of Health under the Secretary of Health Surveillance (34). Weekly influenza activity at the country level across North and South America was obtained from the Global Influenza Programme (GIP) as supported by the World Health Organization, covering 2010 through 2019 (1). To assess seasonal influenza trends, data from 2009—the year of the H1N1 swine flu pandemic—were excluded from all datasets.

Long-run time series of weekly influenza hospitalizations or activity were created at the state (United States, Brazil) and country levels (from the GIP dataset). Locations were included if they had at least one weekly peak of 20 or more cases during the entire time series and if they had at least 2 years of data with more than 15 weeks. We remove location-years with <15 weeks of data when constructing averages. We find that locations with a peak size of <20 cases consisted of very sparse data where it was not possible to construct average case burden. After processing, 81 locations met these inclusion criteria, and weekly average time series were created for each.

### Climate data

#### Historic climate data

Daily temperature and dew point temperature from 1980 to 2015 were downloaded from the ERA5 gridded hourly dataset on single levels with 0.25 ${\;}^{\circ }\times 0.25{\;}^{\circ }$ resolution, representing roughly 30×30 km^2^ grids (Fig. S6) (21). The ERA5 data are created by leveraging various types of observations (eg in situ, radar, satellite, etc.) and weather-forecast model prediction information into a standardized global dataset. It is often treated as “observations” on regular longitude/latitude grids. Specific humidity, a measure of mass of water vapor per unit mass of air, was calculated from surface air pressure (directly from ERA5) as well as saturated water vapor pressure, which is not directly available from ERA5 but derived from dew point temperature using the Magnus form approximation of the Clausius-Clapeyron relation (35). Daily time series spanning the years 1980–2015 for temperature and specific humidity were computed for each analytic location, and the joint distributions of each climate variable per location were plotted (Fig. S7).

#### Climate change projections

Historical simulations (up to year 2014) and future scenario projections (from year 2015 onward) under SSP245 and SSP585 were obtained from 10 CMIP6 models, including: ACCESS-ESM1-5, CESM2, CMCC-CM2-SR5, EC-Earth3-CC, EC-Earth3-Veg-LR, GFDL-ESM4, IITM-ESM, INM-CM4-8, KIOST-ESM, and NorESM2-LM. Daily temperature and specific humidity projections from 2080–2100 (future) and 2000–2015 (historic) were extracted at the second administrative boundary level. To bias-correct the projections, we used the delta-change method where the difference between the historic and future average daily values was calculated and added to the historic ERA5 observations for each location (Fig. S8).

### Mechanistic model with climate

We use a SIRS model incorporating climate as a driver of transmission as proposed by Shaman et al. (2), with modifications for tropical climates as developed by Yuan et al. (18). Specific humidity modifies β(t), the transmission variable, with higher transmission at lower levels of specific humidity. The SIR model is governed by the following equations:


(1) $$\frac{dS}{dt}=\frac{N-S-I}{L}-\frac{\beta (t)I^{a}S}{N}+\mu (N-S)$$

(2) $$\frac{dI}{dt}=\frac{\beta (t)I^{a}S}{N}-\frac{I}{D}-\mu I,$$

where *N* is the population size; *S* is the number of susceptible individuals; *I* is the size of the infected population; *L* is the duration of immunity (years); β(t) is the transmission rate at time *t*; *a* is an exponent that incorporates nonlinearity to approximate population mixing; *μ* is the birth and death rate; and *D* is the duration of infection (years). The β(t) term is related to the basic reproductive number as $R_{0}(t)=\beta (t)D$.


(3) $$R_{0}(t)=[aq^{2}(t)+bq(t)+c]{\left[\frac{T_{c}}{T(t)}\right]}_{\exp}^{T},$$

where *a*, *b*, and *c* are defined in equations 4–6.


(4) $$a=\frac{-b}{q_{\max}+q_{\min}}$$

(5) $$b=\frac{\left(R_{0\max}-(R_{0\max}-R_{0\mathrm{diff}}))(q_{\max}+q_{\min}\right)}{\left(q_{\max}-q_{\mathrm{mid}})(q_{\min}-q_{\mathrm{mid}}\right)}$$

(6) $$c=(R_{0\max}-R_{0\mathrm{diff}})-aq_{\mathrm{mid}}^{2}-bq_{\mathrm{mid}},$$

where $q_{\max}$ represents the specific humidity (g/kg) value at which $R_{0}=R_{0\max}$ when $T=T_{c}$ and is the maximum specific humidity allowed; $q_{\min}$ represents the specific humidity (g/kg) value at which $R_{0}=R_{0\max}$ when $T=T_{c}$ and is the minimum specific humidity allowed; $q_{\mathrm{mid}}$ is the specific humidity at which $R=R_{0\max}-R_{0\mathrm{diff}}$; $R_{0\mathrm{diff}}$ represents the difference between $R_{0\max}$ and $R_{0}$ when $q=q_{\mathrm{mid}}$; $T_{c}$ is the critical temperature below which changes in temperature do not impact transmission; and $T_{\exp}$ determines the strength of the relationship between temperature and $R_{0}$.

### Fitting the SIR model to locations

A range of values were tested to identify best-fit parameters per location based on previous literature, tuning the model to observed influenza data (2, 36–42). The fitted parameters include $R_{0\max}$, $R_{0\mathrm{diff}}$, $q_{\min}$ , $q_{\max}$, $q_{\mathrm{mid}}$, $T_{c}$, $T_{\exp}$, *D*, *L*, $S_{0}$, and $I_{0}$, with optimal parameters identified for each location from the model fit process. Table S1 details the initial parameter ranges, optimized parameter values (including mean and 95% CI), and the weighted average parameter values used for the GAM simulations. A total of 5,000 parameter sets were generated using LHS based on ranges drawn from the literature. For each location, 5,000 simulations were run, and best-fit parameters were selected by minimizing the root mean square error (RMSE) between observed and simulated influenza values. For Fig. 2, an iterative tuning process was used to identify optimal parameters. From the initial 5,000 simulations, the top 500 parameter sets with the lowest RMSE were selected, and a 95% high density interval (HDI) was computed for each parameter. If the upper or lower HDI bound deviated by more than 10% from the original parameter range, the range was updated accordingly. A new set of 5,000 parameter combinations was then generated using LHS and the tuning process was repeated until parameter ranges stabilized within 10% of the 95% HDI bounds. To account for transient dynamics, all simulations were run with a 100-year burn-in period.

Additional specifics on fitting and tuning the climate-driven SIR model are provided in the Supplementary text, including model specification (Table S2).

### EI calculation

EI is defined based on Dalziel et al. as (23):


(7) $$\mathrm{EI}=1-\frac{\sum pln(p)}{ln(\frac{1}{52})},$$

where *p* is a vector of the mean influenza cases or hospitalizations (dependent on data availability) per week divided by the sum across all weeks. EI values were scaled between 0 and 1, as in Dalziel et al.

### Simulating outbreaks under historic and future scenarios

We used our GAM-driven SIRS model to simulate outbreaks at the second administrative level (county-equivalent) across North and South America, under both historic (ERA5) and bias-corrected future (CMIP6 projections) conditions. For each administrative unit, we calculated EI under historic and future scenarios, and differences were computed to quantify climate-driven changes in intensity. This process was repeated across all 10 CMIP6 models, and the median, 10th percentile, and 90th percentile changes in EI were calculated to capture uncertainty across climate change projections. To account for transient dynamics, all simulations were run with a 100-year burn-in period.

## Supplementary Material

pgag160_Supplementary_Data

## Data Availability

All influenza and climate data are publicly available. Code to run the analysis is available at *https://github.com/aleksandrastamper/Unified-clim-factors*. R was used for the statistical computing environment (version 4.4.3) to process, analyze, and visualize data (43).

## Previous presentation

These results were previously presented at Ecology and Evolution of Infectious Diseases in June 2025, and at the Society for Epidemiologic Research in June 2025.
