# Supplementary material for: Unified climate factors predict influenza outbreak seasonality across tropical and temperate regions
Source: PNAS Nexus. 2026 Jun 16;5(6):pgag160. doi: 10.1093/pnasnexus/pgag160 (PMC13271746; doi:10.1093/pnasnexus/pgag160)
Supplement: pgag160_Supplementary_Data [file pgag160_supplementary_data.pdf]

Supplementary Information for: Unified climate factors predict influenza outbreak seasonality across tropical and temperate regions

Stamper et al.

Supplementary Text

Climate-driven model development

Aligning location specific R_0 values

To enable direct comparison of the relationship between specific humidity and R_0 across locations, we aligned R_0 estimates to a common reference location. South Carolina was selected as the reference location as it had the widest specific humidity range. For every other location, we identified the overlapping specific humidity range with that of South Carolina and calculated the mean R_0 for both locations. The difference in mean R_0 was then applied as a vertical offset to shift the entire R_0 curve for the comparison location, aligning its mean R_0 to that of South Carolina within the overlap region. Note, that we do not adjust the shape or range of the locally-fitted R_0 curve. This alignment allowed for a standardized comparison of the specific humidity-R_0 relationships across all locations while controlling for baseline transmission differences. Given the mean R_0 (intercept) of South Carolina may not reflect the best fit values across locations. In later tuning rounds (see Model Tuning) we fit the optimal R_0 intercept that minimizes the RMSE across all locations.

Fitting the generalized additive model (GAM)

A fixed effects GAM was fit to estimate logged influenza activity (cases or hospitalizations, dependent on data availability) as a function of specific humidity, with fixed effects at the week, year, and location (equation 8). The GAM was fit to both temperate and tropical location data.

$$\begin{aligned}\log(\text{influenza activity}) \sim s(q_t, k=3) + \text{factor(week)} \\ +\text{factor(year)} + \text{factor(location)}\end{aligned} \tag{1}$$

Where $s(q_t,\ k=4)$ is a thin plate spline for weekly mean specific humidity and there are fixed categorical effects for the week number to account for seasonality.

A GAM was fit to estimate the basic reproduction number as a function of specific humidity and temperature (equation 9).

$$R_0(t) \sim s(q_t,\ k = 5) + s(T_t,\ k = 5) \qquad (2)$$

Where $s(q_t\ k = 5)$ is specific humidity modeled as a smooth spline term using a thin plate linear regression spline with 5 knots and $s(T_t,\ k = 5)$ is temperature modeled as a smooth spline term using a thin plate linear regression spline with 5 knots. The model was fit using restricted maximum likelihood (REML).

The GAM was fit to the climate-transmission estimates shown in Figure 3A. As the q_{min} parameter reflects the specific humidity threshold at which $R_0 = R_{0max}$, retaining values below it resulted in an artificial ledge at lower levels of specific humidity. While this ledge was helpful for visualizing the low specific humidity cutoffs for each location, fitting a GAM to data with specific humidity values below q_{min} resulted in a worse fit when aggregating data across multiple locations (Supplementary Figure 2A, 2B). As such we remove the q_{min} plateau when fitting the GAM.

Linking GAM to the SIRS model

Next, we integrated the GAM-estimated transmission surface into the SIRS model to simulate influenza outbreaks. We used the GAM to capture all climate-related parameters (R_{0max}, R_{0diff}, q_{min} , q_{max}, q_{mid}, T_c, T_{exp}), which were previously dependent on the individual model fits. The remaining non-climate parameters ($D,\ L,\ S_0$, and I_0) were estimated as weighted averages of their original best-fit values across locations, where weights reflected the goodness-of-fit of the original SIRS model at each location. To improve SIRS-GAM model fit to the observed data, we underwent an iterative tuning process along two axes: (1) global intercept selection; and (2) GAM model specification (i.e., adding additional factors into the GAM). For each intercept-specification combination, we ran the SIRS model for all locations and calculated the root mean squared error (RMSE) to identify the optimal configuration.

Model tuning

We apply an iterative tuning process to improve the fit of the GAM-driven SIRS model to observed influenza cases. Several GAM options were evaluated, including models where R_0 is influenced by the mean and range of the climate factors. Supplementary Table 2 displays results from the GAM fit process, highlighting the model specification resulting in the lowest RMSE across all locations. We find the best fit is given by:

$$R_0(t) \sim s(q_t,\ k=5) + s(T_t,\ k=5) + \text{Trange}_l + \text{Qrange}_l \tag{3}$$

Where $s(q_t\ k=5)$ is specific humidity modeled as a smooth spline term using a thin plate linear regression spline with 5 knots, $s(T_t,\ k=5)$ is temperature modeled as a smooth spline term using a thin plate linear regression spline with 5 knots, and local temperature range ($Trange_l$) and specific humidity range ($Qrange_l$) were included as linear terms. The model was fitted using restricted maximum likelihood (REML).

We also optimize the R_0 intercept which is combined with the GAM-dependent climate term to provide the location-specific R_0 profile. We do this is two ways: fitting a location-specific intercept γ_i or a global intercept γ_g. We then re-run our SIRS model using fitted γ_i and γ_g separately to compare global fit metrics (Fig. 3B). While location-specific intercept factors minimize the RMSE, we find comparable RMSE using a global intercept term which we then use to run the model for out-of-sample locations.

Supplementary Figures

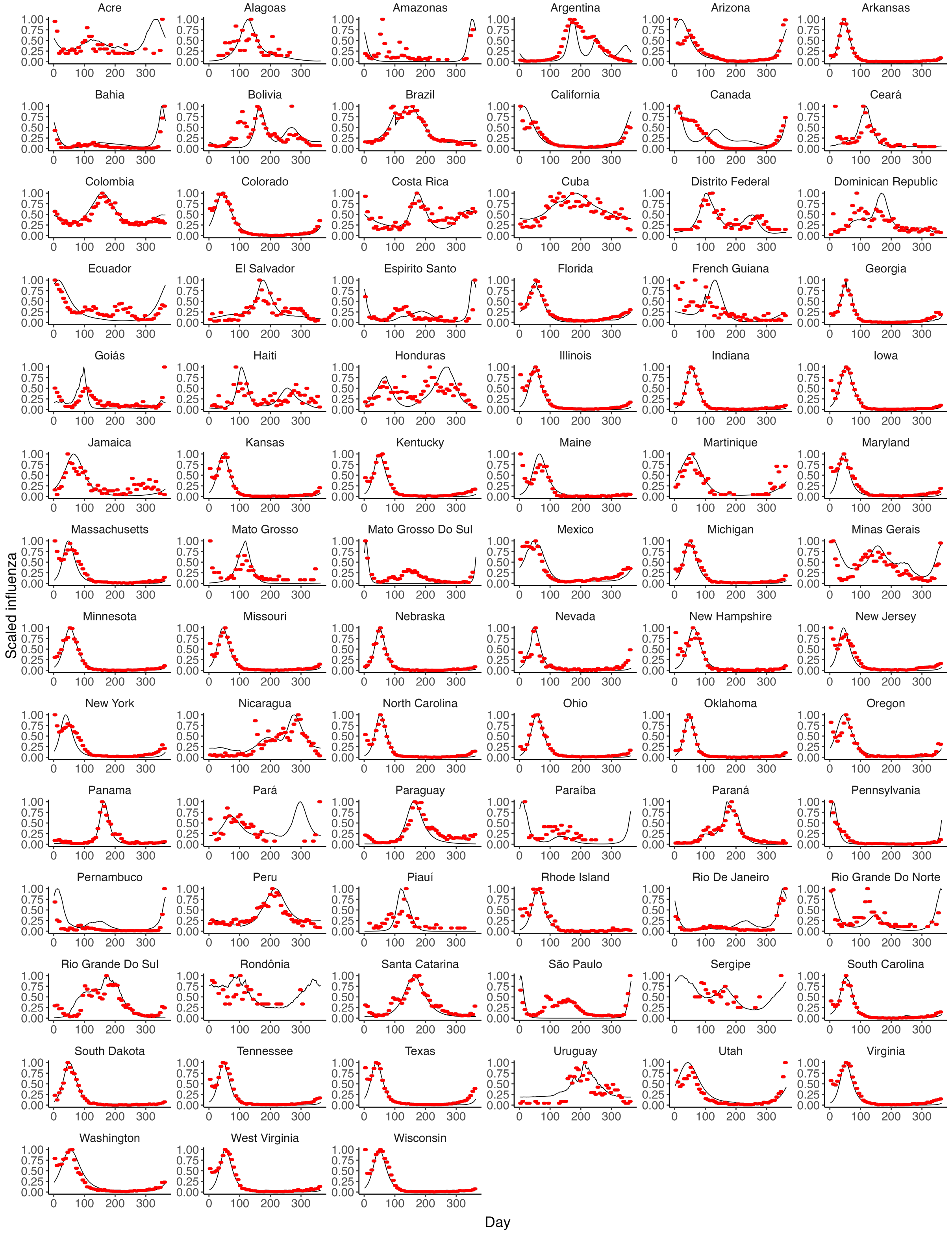

Figure 1: Best-fit model for locations across North and South America, national and sub-national locations. Red points indicate observed values and black line indicates model simulation.

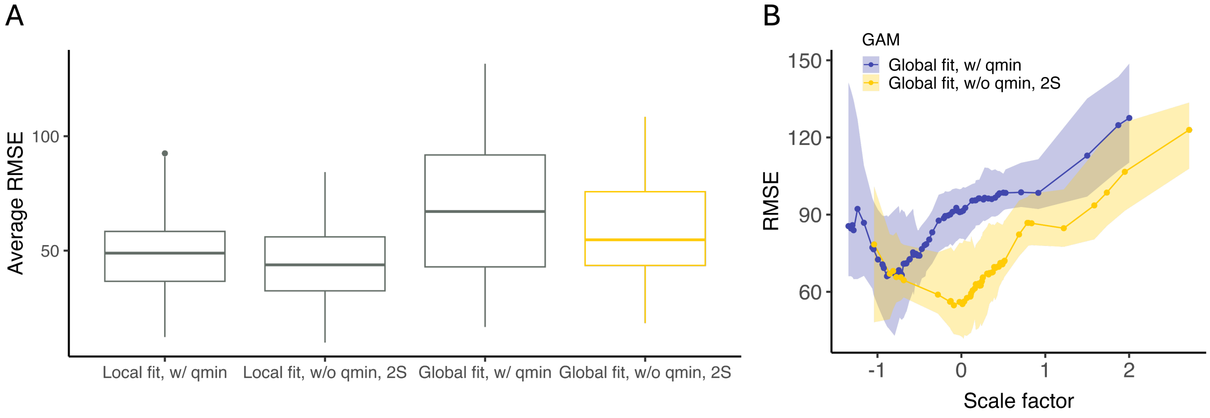

Figure 2: GAM selection process (testing global intercept values and model specification) to identify a model configuration that minimizes error between observed and simulated influenza outbreaks. **A.** Assessing RMSE ranges for two GAMs, where local fit indicates the best-fit for each individual location, and global fit indicates the best-fit scaling factor for all locations. **B.** Comparing the RMSE ranges for both GAMs at each scaling factor tested.

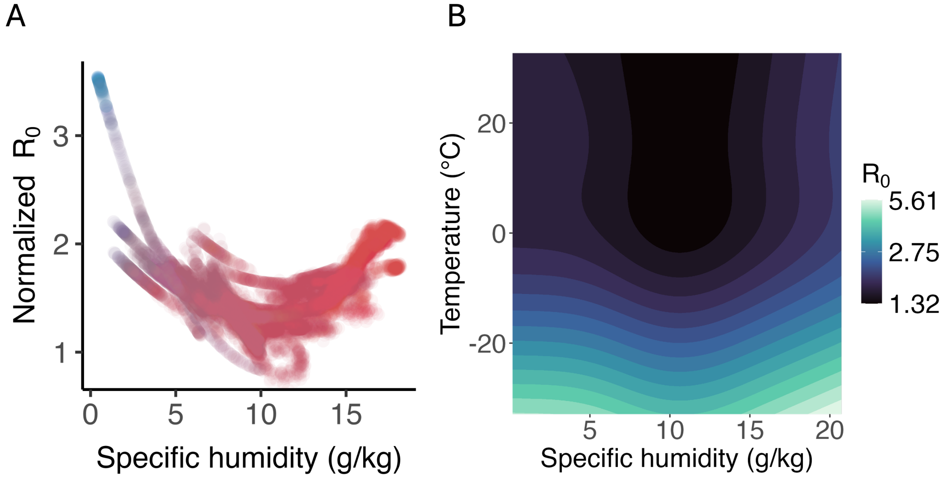

Figure 3: Estimated effects of temperature and specific humidity on influenza transmission. **A.** GAM indicating combined effects of specific humidity, $q(t)$ (g/kg), and temperature, $T(t)$ (C), on R_0. **B.** Surface plot of predicted influenza transmission values derived from the observed ranges of temperature and specific humidity.

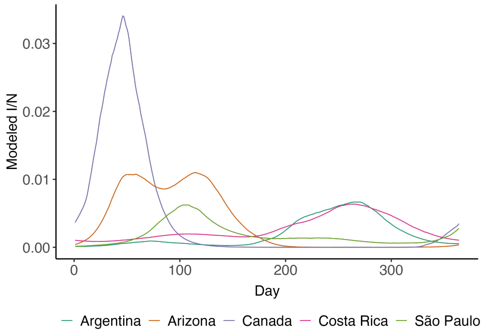

Figure 4: Projected I/N in sites referenced in main Figure 3D (Argentina, Arizona, Canada, Costa Rica, and São Paulo) ranging from temperate to tropical climates using the GAM to estimate R_0.

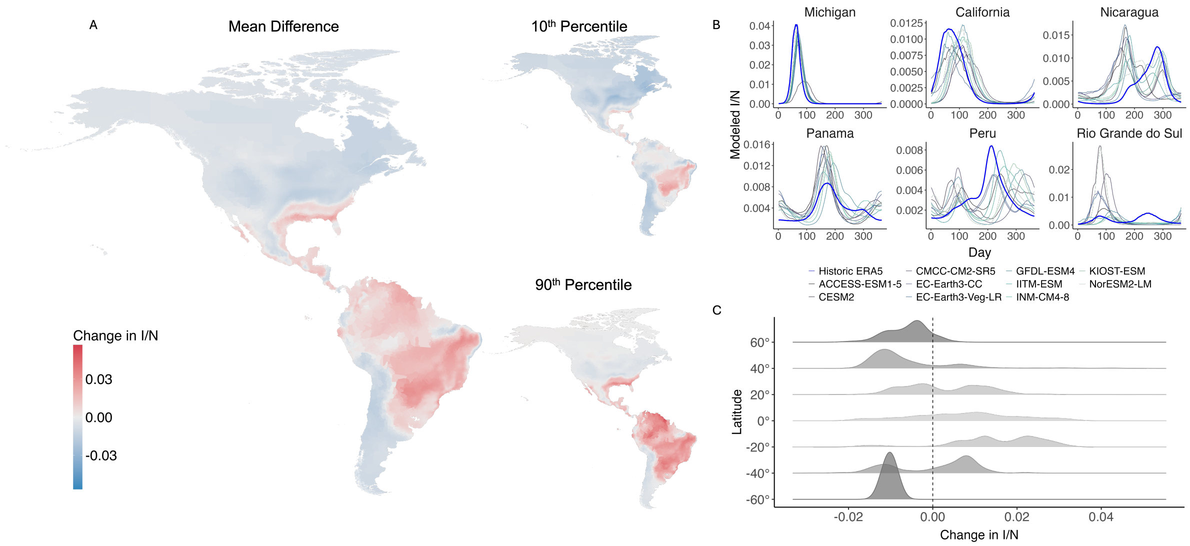

Figure 5: Simulated influenza outbreaks under historic and future climate projections across North and South America. **A.** Estimated change in the maximum infected peak size comparing historic to mean, 10th percentile, and 90th percentile SSP585 projections for 2080-2100. **B.** Influenza time series comparing historic to average SSP585 climate change projections for 2080-2100. **C.** Ridgeline plot showing change in maximum infected peak categorized into 20-degree latitude bins.

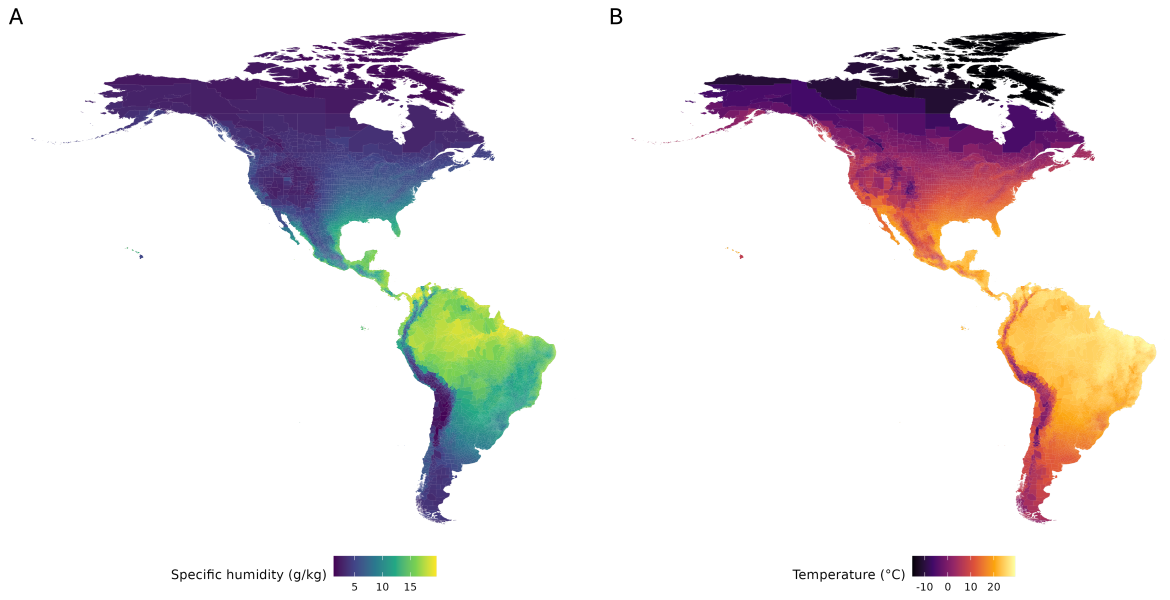

Figure 6: Plots showing historic (A) specific humidity (g/kg) and (B) temperature (C) across North and South America at the second administrative level, averaging daily values across 1980-2015 from ERA5 data.

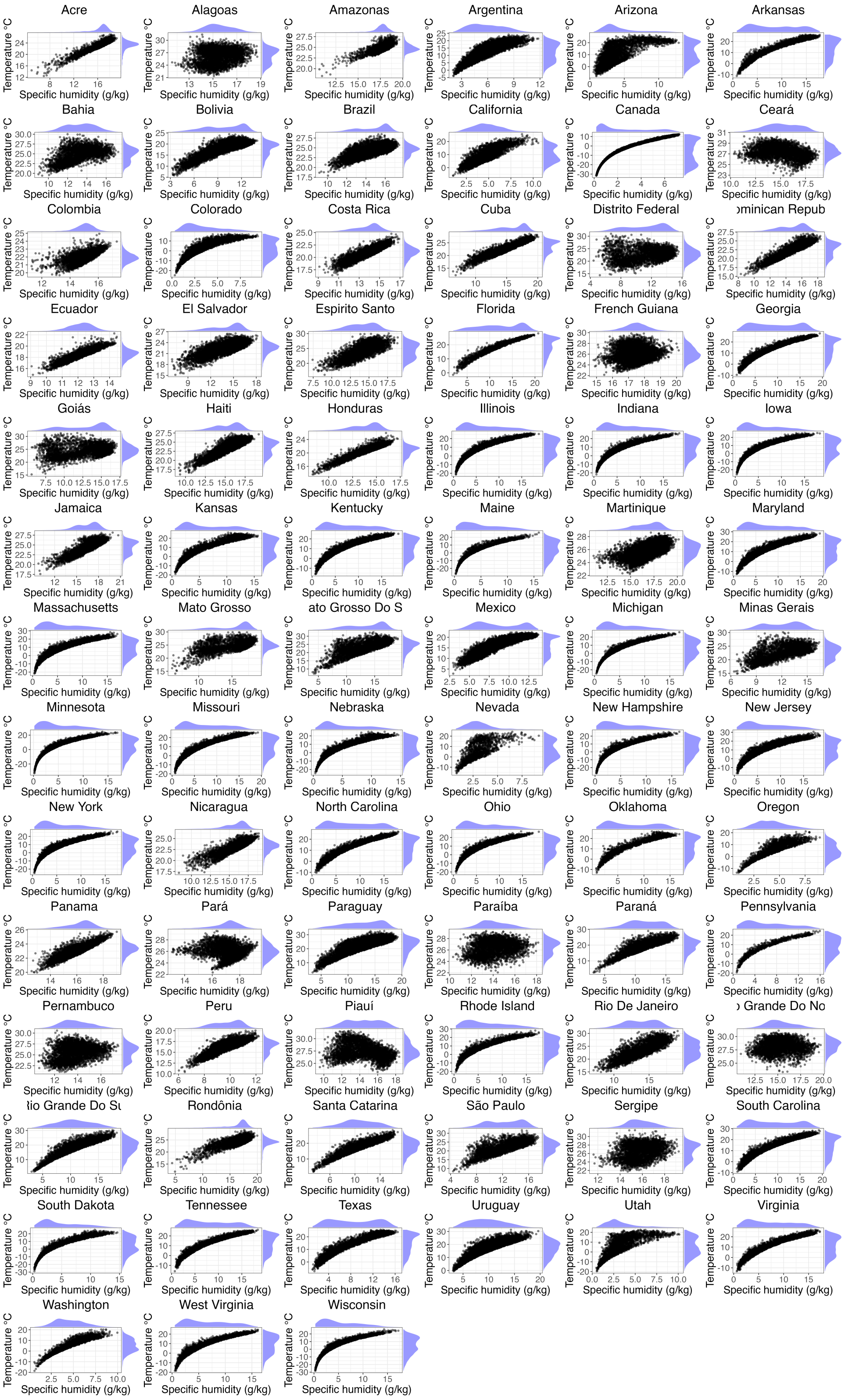

Figure 7: Joint distribution of specific humidity (g/kg) and temperature (C) for each training location. Climate variables indicate average daily values from 1980-2015 from ERA5 data.

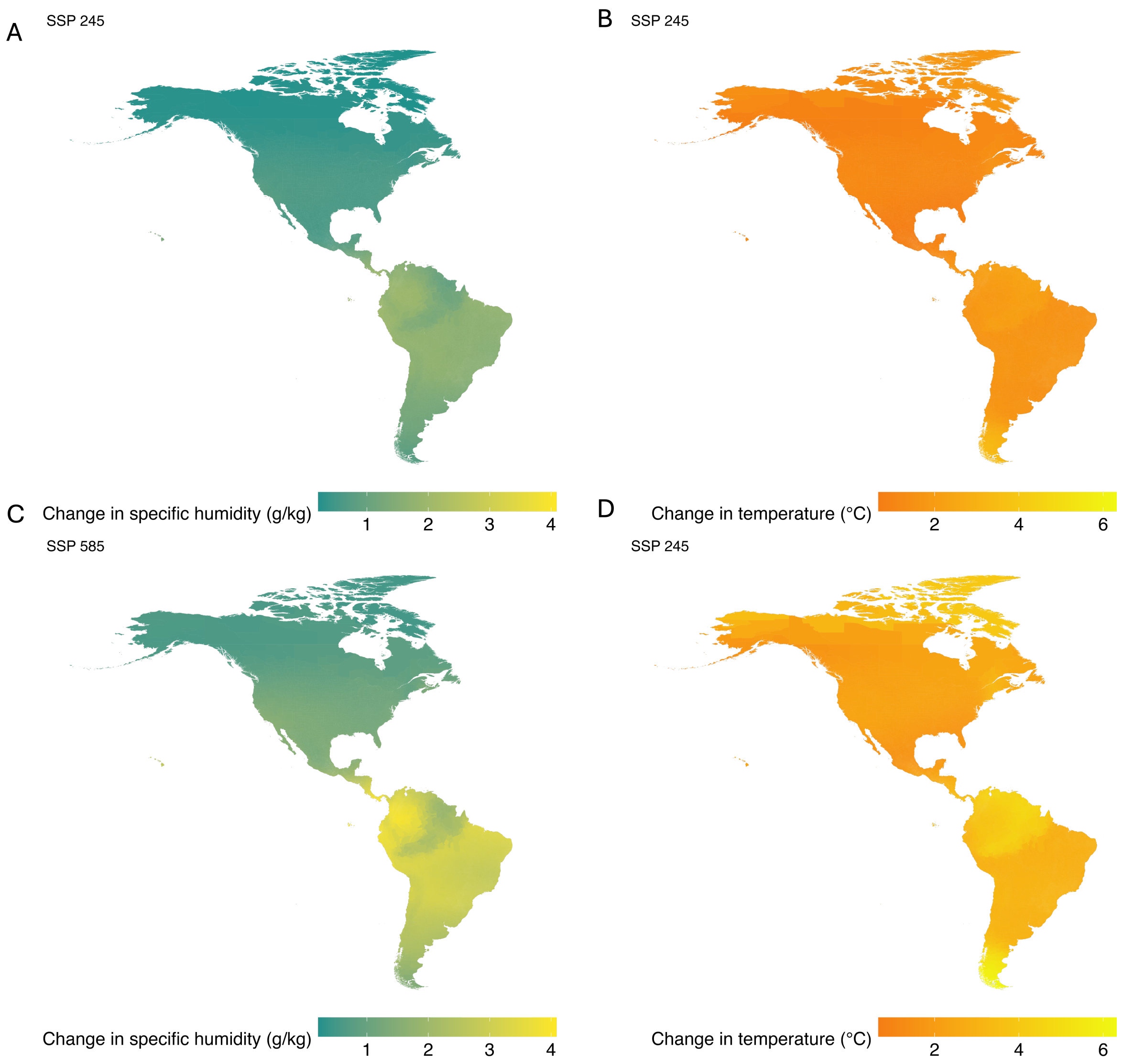

Figure 8: Average changes in specific humidity and temperature comparing ten CMIP6 models to ERA5 data at the second administrative level across North and South America under two climate change scenarios. Average change in specific humidity under SSP245 (**A**) and SSP585 (**C**), and average change in temperature under SSP245 (**B**) and SSP585 (**D**).

Supplementary Tables

Table 1: Initial parameter ranges, optimized parameter values (mean, 95% CI), and weighted optimal parameters used for GAM-driven SIRS simulations.

**Parameter**	**Initial parameter range**	**Optimized parameter values (mean, 95% CI)**	**Weighted optimal parameters (for GAM)**
S_0	0.4 – 0.8	0.613 (0.581, 0.646)	0.6087966
I_0	5000 – 1,000	941 (866, 1,016)	879.4905
D	2–5	3.68 (3.41, 3.95)	3.693802
L	365 – 3,650	436 (427, 445)	437.3574
q_{min}	17 – 20	3.77 (3.00, 4.54)	–
q_{mid}	0 – 8	11.5 (11.3, 11.8)	–
q_{max}	4 – 12	19.0 (18.4, 19.6)	–
$R_{0\,\text{max}}$	1.5 – 3	2.11 (1.97, 2.24)	–
$R_{0\text{diff}}$	0.86 – 1.18	0.890 (0.840, 0.941)	–
T_c	20.4 – 30	23.1 (22.7, 23.4)	–
T_{diff}	0 – 15	4.01 (2.93, 5.10)	–
T_{exp}	0.95 – 1.54	1.08 (0.970, 1.200)	–

Table 2: Model tuning results where RMSE values are for fitting the GAM-driven SIRS model to influenza case data across location. We find the best fitting GAM model includes average specific humidity range and temperature range.

Model specification	Mean RMSE	Median RMSE
$R_0(t) \sim s(q_t,\ k = 5) + T_{t,l}$	73.7	70.9
$R_0(t) \sim s(q_t,\ k = 5) + s(T_t,\ k = 5)$	73.7	70.9
$R_0(t) \sim s(q_t,\ k = 5) + s(T_t,\ k = 5) + \text{Tavg}_l + \text{Qavg}_l$	73.6	70.8
$R_0(t) \sim s(q_t,\ k = 5) + s(T_t,\ k = 5) + \text{Trange}_l + \text{Qrange}_l$	72.6	70.0
